# The Otitis Media-6 questionnaire: psychometric properties with emphasis on factor structure and interpretability

**DOI:** 10.1186/1477-7525-11-201

**Published:** 2013-11-20

**Authors:** Christian Hamilton Heidemann, Christian Godballe, Anette Drøhse Kjeldsen, Eva Charlotte Jung Johansen, Christian Emil Faber, Henrik Hein Lauridsen

**Affiliations:** 1 Department of ENT Head & Neck Surgery, Odense University Hospital, Odense C 5000, Denmark; 2 Institute of Clinical Research, Faculty of Health Sciences, University of Southern Denmark, Odense M 5230, Denmark; 3 Ear Nose Throat Private Clinic, Odense C 5000, Denmark; 4 Research Unit for Clinical Biomechanics, Institute of Sports Science and Clinical Biomechanics, University of Southern Denmark, Odense M 5230, Denmark

**Keywords:** Cross-cultural adaptation, Validation, OM-6, Otitis media, Factor analysis, Minimal important change, Smallest detectable change

## Abstract

**Background:**

The Otitis Media-6 questionnaire (OM-6) is the most frequently used instrument to measure health related quality of life in children with otitis media. The main objectives of this study are 1) to translate and cross-culturally adapt the OM-6 into Danish, and 2) to assess important psychometric properties including structural validity and interpretability of the OM-6 in a Danish population of children suffering from otitis media.

**Methods:**

The OM-6 was translated and cross-culturally adapted according to international guidelines. A longitudinal validation study enrolled 491 children and their families, and the measurement properties of the OM-6 were evaluated using the Cosmin taxonomy. The properties assessed were construct and structural validity (confirmatory factor analysis) including internal consistency, reproducibility (test-retest reliability and smallest detectable change), responsiveness and interpretability.

**Results:**

A total of 435 children were eligible to participate in the study. Analyses of structural validity and internal consistency indicated that parent appraisal of hearing and speech problems may be problematic. Both scales showed similarly good test-retest reliability and construct validity, were able to discriminate between diagnostic subgroups and responsive to change. Cut-off values of 16.7 and 30.0 were found to represent minimal important change for the patients.

**Conclusions:**

The Danish version of the OM-6 is a reliable, valid, responsive and interpretable questionnaire to measure quality of life in children with otitis media. This study sheds light on possible weaknesses of the instrument that needs to be acknowledged in the utilization of the instrument. However, despite these issues our results support the continuing use of OM-6 as a 1-factor functional health scale with a separate global health rating. Furthermore, indications of values representing minimal important change as perceived by the respondent are presented.

## Introduction

Otitis media (OM) is a common childhood disease and the leading cause of doctor consultations for pre-school children [1]. It can be divided in two major diagnostic subgroups: acute otitis media (AOM) and otitis media with effusion (OME) with great overlap between the two (see Table 1) [2-4].

**Table 1:** Definitions of diagnostic subgroups of otitis media

AOM:	Middle ear effusion and acute onset of signs and symptoms of middle ear inflammation such as fever, otalgia, possible otorrhoea and discomfort that may result in interference with or precludes normal activity or sleep [3].
	Recurrent acute otitis media (rAOM) is defined by the presence of at least 3 episodes of acute otitis media in 6 months or 4 or more episodes in 1 year [4].
OME:	Middle ear effusion without signs or symptoms of acute ear infection [2]. Disease severity of OME ranges from no symptoms to lowered activity level and sleep disturbances or even significant hearing loss and speech impairment.

Quality of life (QoL) as an outcome for assessment of treatment has become increasingly recognized in clinical research and several studies have assessed the quality of life of children with otitis media. Since the development of the disease specific proxy-completed Otitis Media-6 (OM-6) questionnaire in 1997 [5], it has become the most frequently used questionnaire applied in the international literature [6,7]. The questionnaire covers physical and emotional domains of functional health status (FHS) in the child and concerns of the caregiver. Furthermore, it includes a measure of global health related to child quality of life. The questionnaire can be completed within a matter of minutes making it ideal for application in different settings.

Successful application of a QoL measure in clinical research is not only dependent on the study design but also on the psychometric properties of the included outcome measures. The literature on assessment of the psychometric properties of OM-6 is limited to a few studies [5,8-11] and several important aspects of the psychometric quality of the instrument have yet to be investigated. For example, no studies have reported on analysis of the factor structure of OM-6 which is fundamental to the analysis of validity. In addition, specific analyses of measurement error, smallest detectable change or respondent perceived minimal important change are absent – parameters important for interpretability of the OM-6.

The main objectives of this study are 1) to translate and cross-culturally adapt the OM-6 into Danish, and 2) to assess important psychometric properties including structural validity and interpretability of the OM-6 in a Danish population of children suffering from OM.

## Methods

### Patients and design

Children and their families were consecutively enrolled in the study from 13 private Ear Nose Throat (ENT) clinics on the island of Funen, Denmark from February 15th 2011 to February 28th 2012 as part of a larger cohort study investigating the impact of ventilating tube treatment on the quality of life of the child and caregiver. Inclusion criteria were 1) indication for bilateral or unilateral ventilating tube treatment established by an ENT specialist, 2) age 0–6 years, 3) no history of previous ventilating tube treatment, 4) caregiver should be able to read, write and understand Danish. Exclusion criteria were syndrome diseases, cleft lip and palate or other concurrent illnesses with the potential to affect the quality of life such as severe heart or lung disease.

The study design was purely observational and approval from the local ethics committee was not required according to the rules and regulations of the Danish scientific ethical committee. However, the study was reported to and accepted by The Danish Data Protection Agency.

### Measurement tools

#### Otitis media-6 questionnaire

The OM-6 includes 6 FHS items measuring the child’s physical suffering, hearing loss, speech impairment, activity limitations, emotional distress and caregiver concern and a numerical rating scale (NRS-child) for the assessment of global QoL in the child. Respondents (parents) are asked to recall symptom history pertaining to the previous four weeks. Each of the FHS items is scored on a Lickert-type scale ranging from 1 to 7, with 7 representing worst score. The NRS scale ranges from to 0 to 10, with 0 representing worst score. Originally, the summary score of the FHS scale was produced directly by summing up the scores of the 6 items and dividing by the total number of items [5], but in later work the developers have adjusted the item scores to 0–100 scales before dividing by the total number of items [12]. We adopted the latter method but computed the summary score based on the method of proportional recalculation [13]. The items were adjusted to a scale of 0 to 100, with 0 as “no impact” and 100 as “worst possible impact”. The summary score was then determined by the mean of items that were answered, rather than just the total number of items. The NRS scale ranges from to 0 to 10, with 0 representing worst score. For comparability this scale was also adjusted to a 0 to 100 scale.

The principles of forward-backward translation were applied to the OM-6 in accordance with the guidelines proposed in the literature [14]. Written consent to translate and apply the questionnaire was obtained from the original developers.

#### Additional measurement tools

Additional instruments were included for the purpose of validation. The Child Health Questionnaire - CHQ-PF50 is a widely used generic questionnaire for measuring QoL in children. It is available in a validated Danish version. The following subscales where included: Global Health, Role/Social limitations – Physical, Bodily Pain/Discomfort, Mental Health, General Health Perceptions, Parental Impact – Emotional and Parental Impact – Time) [15-20]. Caregiver Impact Questionnaire (CIQ) is a disease specific instrument developed to assess the impact of otitis media on caregiver QoL [12]. The structure of CIQ is similar to OM-6. The CIQ is only available in a validated English version. It has been translated and validated by our research group (awaiting publication). Furthermore, four questions regarding the number of doctor visits, days of antibiotic use, days of observed lower activity level in the child and interrupted nights of the caregiver because of OM in the child. Lastly, a seven point global perceived effect (GPE) scale in which parents stated to which extend they had experienced changes in the disease specific QoL of the child after intervention. The seven response options were: 1) very much improved, 2) much improved, 3) a little improved, 4) no change, 5) a little deterioration, 6) much deterioration, 7) very much deterioration.

### Procedure

Questionnaire booklets were administered to the families at three time points. Parents were asked to complete the questionnaires on the day the ENT specialist established indication for ventilating tube treatment (pre-baseline), within four days prior to surgery (baseline) and one month post-surgery (follow up). All pre-baseline questionnaires were handed out and completed on paper. For all subsequent questionnaires caregivers were given the choice between paper based questionnaires or electronic questionnaires. Eighty-two percent of caregivers chose to complete all subsequent questionnaires online. We regarded respondents who completed the baseline questionnaire more than 7 days after ventilating tube treatment as not eligible for data analysis. The following questionnaire booklets were handed out at the three time points. At pre-baseline OM-6 and CIQ were included. At baseline and follow-up the booklet consisted of OM-6, CIQ and all additional measurement tools as mentioned above.

### Statistical analysis

The Cosmin taxonomy for measurement properties were applied as a basis for the statistical analysis of this study [21,22].

#### Missing items

Missing items were investigated at baseline and follow up. If more than 50% of items were missing, the form was discarded (regarded as missing in total). Less than three percent missing scores on each item was considered acceptable [23].

#### Validity

##### Structural validity and internal consistency

Confirmatory factor analysis (CFA) was conducted in order to investigate the accuracy of the hypothesized one factor structure of OM-6 [5]. Investigations on the structure of OM-6 are warranted for different reasons. Despite its brevity the instrument covers both emotional and physical domains of FHS which renders a more complex structure possible. Furthermore, it is designed to cover diagnostic subgroups (rAOM and OME) of which the clinical picture may vary greatly and the factor validity is likely to differ between these diagnostic subgroups. Asymptotically distribution free estimation was applied because of non-normality. Model accuracy was based on the chi-square test and the following model fit indices: 1) comparative fit index (CFI), 2) root mean square error of approximation (RMSEA), and 3) standardized root mean square residual (SRMR). As the relatively large sample size has the potential to produce statistically significant chi-square values which are essentially unimportant, all significant chi-square values were interpreted in combination with the other fit indices [24]. Model fit was interpreted as ‘acceptable’ if CFI >0.90, RMSEA <0.08 and SRMR <0.08. Model misspecifications were calculated using the expected parameter changes (EPC) and modification index (MI) [25]. Secondly, internal consistency was assessed by calculating Crohnbach’s alpha and item-total correlations. Alpha should be between 0.70 and 0.95 [26].

##### Construct validity

Hypotheses were constructed regarding correlations between items (inter-item), between items and summary scores (item-total) and between summary scores (total-total) within and between the questionnaires [23,26]. A higher percentage of correct predictions indicate stronger support for construct validity. A correlation of <0.3 was defined as weak, 0.3-0.5 as moderate and >0.5 as strong [27]. Negative correlations were expected between the FHS scale of OM-6 and the NRS scales of OM-6 and CIQ and the subscales of CHQ-PF50 as these are inversely scored. Wilcoxon rank-sum (Mann–Whitney) test was used to test if the scales were able to discriminate between diagnostic subgroups.

#### Reproducibility

##### Test-retest reliability

Reliability was assessed by calculating the intraclass correlation coefficient (ICC2.1.A) [28]. Criteria for inclusion in the test-retest analysis were 1) because OM is a fluctuating disease only repeated measurements with an interval of 2 to 14 days between pre-baseline and baseline measurements were included, 2) caregivers had to state that they perceived the state of OM in their children to be static between the repeated measurements and 3) the respondent should be the same at both measurements. An ICC of at least 0.70 is required as a minimum standard for test-retest reliability [26,29,30].

##### Smallest detectable change (SDC)

SDC is based on Standard Error of Measurement (SEM) which is the variability in measurements (SD) of the same individual with a confidence of 95% and is expressed in the unit of the measurement. It was estimated by computing the square root of the within subject variance of the patients (SEM_agreement_ = √σ_between measurement_ + σ_residual_) [22]. Variance components were obtained from a multilevel mixed effects model (restricted maximum likelihood estimates) [31]. Because SDC is the smallest amount of change in individuals that can be detected beyond measurement error with a confidence of 95%, it is calculated as 1.96*√2*SEM [26].

#### Responsiveness

##### Criterion responsiveness

A GPE scale was used as an external anchor in the assessment of criterion responsiveness. Because it was considered a gold standard of measuring change, it was hypothesized that correlations between change scores and GPE scores should be at least 0.5 [32]. Subsequently, receiver operating characteristic (ROC) analyses were performed in which respondents were dichotomized according to their responses on the GPE-scale. We considered patients choosing response option 1–2 (“very much improved” and “much improved”) on the GPE-scale as “importantly improved” and those choosing option 3–5 (“a little improved”, “no change” and “a little deterioration”) as “stable”. The area under the curve (AUC) was interpreted as the probability of correctly identifying the “importantly improved” children from “stable” children. An AUC of 1.00 indicates perfect discrimination whereas an AUC of 0.50 indicates that discrimination is no better than chance. AUC should be at least 0.70 [26].

##### Construct responsiveness and floor/ceiling effects

Construct responsiveness was assessed by hypothesizing that correlations between change scores of the different questionnaires would be at least 0.5 [23,26]. Lastly, Floor and ceiling effects were investigated at baseline as presence of these may hamper the possibility of detecting change. These were considered present if more than 15% achieved the highest or the lowest possible score [26,33].

#### Minimal important change (MIC) as perceived by the respondent

For determining the MIC we used a three step procedure that integrates both anchor-based and distribution-based methods [34]. In step one the study sample is dichotomized according to the anchor in groups of “importantly improved” children versus “stable” children as described in the paragraph on criterion responsiveness. In step two the distribution of change scores is plotted. Proportional frequencies are used in order to avoid influence of the sample size of the groups on the curve and cut-off points. In step three the MIC is determined by using the optimum ROC-point cut-off point as benchmark. By weighting sensitivity and specificity equally this cut-off point is assumed to represent the lowest overall misclassification. Lastly, the MIC was related to the SDC by computing the group size needed to achieve an SDC_group_ that equals the MIC (n = (SDC/MIC)^2^) [35].

STATA® v. 12.1 IC (StataCorp) was used for all analyses.

## Results

### Translation and cross-cultural adaption of OM-6

The translation and cross-cultural adaptation of the OM-6 resulted in several pertinent issues. The term “ear infection” was changed to the more specific “middle ear infection” to minimize the risk of including infection of the external ear canal and auricle. Semantic adaption of some items was necessary either because a direct translation rendered incomprehensible sentences or the risk of respondent misinterpretation was high. For example “suffering” in item 1 was changed from “lidelse” (suffering) to “gener” (bothersomeness) as “lidelse” (suffering) is a very strong expression in Danish. Similarly, “hearing loss” in item 2 was changed from “høretab” (total hearing loss) to “nedsat hørelse” (reduced hearing), to avoid the risk of respondents interpreting the wording as a total loss of hearing. Finally, “caregiver” which is used in item 6, was changed to “forælder” (parent) for two reasons; “caregiver” is not commonly used in the Danish language and “parent” is often used in the meaning of “caregiver”.

### Sample population and scale descriptives

Four-hundred-ninety-one families were enrolled in the study. Fifty-six had to be excluded because of late baseline responses (> 7 days post-surgery). Response rates were 95.4% and 92.6% at baseline and follow up, respectively. Missing items ranged from 0.0-1.6% for the 6 FHS items with only items 2 (1.1%) and 3 (1.6%) having more than 0.5%. Basic demographic data and scale scores are presented in Table 2.

**Table 2:** Sample demographics and scale scores

	**Total sample**	**OME**	**rAOM**	**rAOM/OME**
Gender, no (%)*				
Male	244 (56.1)	115 (55.8)	35 (55.6)	92 (56.4)
Female	191 (43.9)	91 (44.2)	28 (44.4)	71 (43.6)
Age at surgery*, median (iqr)^a^	1.46 (1.23)	1.77 (2.10)	1.22 (0.85)	1.37 (0.72)
Baseline scores, mean (SD)**				
FHS	44.5 (18.9)	39.5 (18.7)	51.1 (16.8)	48.4 (18.3)
NRS-child	49.6 (23.2)	56.6 (24.0)	41.8 (18.7)	44.0 (21.2)
Follow up scores, mean (SD)***				
FHS	17.7 (15.6)	17.4 (16.0)	16.8 (17.4)	18.5 (14.4)
NRS-child	80.6 (18.6)	81.3 (18.0)	81.5 (19.5)	79.3 (19.2)

*N=435 - information on diagnostic subgroup missing for 3 children, **N=415, ***N=403.

^a^interquartile range.

### Validity

CFA revealed issues in the hypothesized one factor structure of OM-6. A poor fit was obtained from the initial analysis and modification indices (MI) gave indications to possible changes of the model. Error terms of item 2 (hearing loss) and item 3 (speech impairment) correlated highly which made conceptual sense. A shared covariance of these items was included in the analysis and a superior fit was obtained. Subsequently, data was dichotomized into groups of children experiencing recurrent episodes of AOM with or without OME (+rAOM) and those who had only experienced OME (-rAOM). Our modified model fitted well on the data from the latter subgroup. However, further model modifications were necessary to obtain an acceptable fit on the data from the group experiencing rAOM or rAOM/OME. Large correlation was found between error terms of item 4 (emotional distress) and item 6 (caregiver concerns) which also made conceptual sense. An acceptable fit was obtained after including a shared covariance of these items. Fit statistics are presented in Table 3. Internal consistency of the one factor model was acceptable (alpha = 0.75) and item-total correlations ranged from 0.46-0.79. Only two items had correlations below 0.7 (item 2: 0.50 and item 3: 0.46).

**Table 3:** Confirmatory factor analysis model fit

	**Total sample**		**Subgroup analysis**	
	**Original model**	Final modified model*	-rAOM*	+rAOM**
N	400	400	191	209
Chi^2^	153.720	25.290	9.722	16.477
d.f.	9	8	8	7
*p-value*	>0.001	0.001	0.285	0.021
CFI	0.687	0.963	0.992	0.958
RMSEA	0.200	0.074	0.034	0.080
SRMR	0.153	0.036	0.036	0.045

*covariance of item 2 and 3 included in the model, **covariance of item 2 and 3 and covariance of item 4 and 6 included in the model.

Construct validity was assessed by testing 24 hypothesized correlations. Table 4 provides examples of hypotheses (full list available in Additional file 1) and Table 5 displays number of correctly and incorrectly predicted correlations. Twenty-one (87.5%) hypothesized correlations were correct. Furthermore, both scales were able to discriminate between children suffering from rAOM and children suffering from only OME (Table 5).

**Table 4:** **Examples of construct validity hypotheses (full list available in Additional file****1**)

	**Correlated to**			
	**Questionnaire (subscale or item number)**	**Hypothesized correlation**	**Comment**	**Obtained correlation**^**a**^
FHS (phys. suffering)	CHQ-PF50 (bodily pain)	Strong, negative	If pain is present in OM, it will also become apparent in more generalized questions.	-0.82
FHS (activity limitations)	No. of days observed lower activity level	Strong, positive	Both items regard the child’s activity level, although OM-6 item is more extensive.	0.56
NRS-child	CHQ-PF50 (Global health)	Moderate, negative	Global health is likely to be affected by disease specific QoL.	-0.33
FHS (summary score)	CIQ FHS summary score	Strong, positive	Child FHS has a strong influence upon caregiver FHS.	0.72

^a^Spearman’s rho.

**Table 5:** Construct validity – matrix displaying results on the different analyses of construct validity

	**Convergent and discriminant validity**		**Discriminative validity**		
	**Correctly predicted**	**Incorrectly predicted**	+ rAOM* mean (SD)	- rAOM** mean (SD)	***p-value***^**a**^
FHS	16	3	49.1 (17.9)	39.5 (18.7)	<0.001
NRS-child	5	0	43.4 (20.5)	56.6 (24.0)	<0.001

*N = 216, **N = 197.

^a^Wilcoxon rank-sum (Mann–Whitney) test.

### Reproducibility

Data from 135 respondents were included in the reproducibility analysis. There was a mean of 6.7 days between the measurements with only small and non-significant differences between this subsample and the remaining study sample with regards to age, gender, diagnostic distribution and baseline scores (Additional file 1). ICC was acceptable for both scales (OM-6: ICC 0.85, CI 0.80-0.89, NRS: ICC 0.83, CI 0.77-0.88). The mean difference was close to zero indicating no systematic difference between test-retest scores and SDC was relatively large for both scales (OM-6: 19.7, NRS: 25.9). This means that a change of less than one fifth of the whole scale cannot be detected beyond measurement error on the individual level (Table 6).

**Table 6:** Reproducibility–test-retest reliability and smallest detectable change

	**N**	**Difference score (SD)**^**a**^	**ICC (CI)**	**SEM**	**SDC**
FHS	135	1.3 (10.0)	0.85 (0.80-0.89)	7.1	19.8
NRS-child	135	0.6 (13.2)	0.83 (0.77-0.88)	9.3	25.9

^a^Difference between test-retest scores.

### Responsiveness

Three-hundred-and-ninety-seven caregivers completed both baseline and follow-up questionnaires. Criterion responsiveness hypotheses regarding correlations between change scores and GPE score were only confirmed for the FHS scale. However, hypotheses for the AUC were confirmed for both scales (Table 7). Construct responsiveness was found to be good as change scores of the instruments correlated well with each other (Table 7). As anticipated, correlations between change scores of the disease specific questionnaires were higher than correlations to the generic CHQ-PF50 questionnaire. On the FHS summary score 0.5% and 0.0% scored the lowest and highest possible scores, respectively whereas this was 0.7% and 2.2% on the NRS score.

**Table 7:** Responsiveness – construct and criterion responsiveness

	**Change score mean (SD)**	**Construct responsiveness**					**Criterion responsiveness**	
		**Correlation**^**a**^					**Correlation**^**a**^	**ROC-analysis**
		**CHQ-PF50**^**b**^	**FHS**^**c**^	**NRS-child**^**c**^	**CIQ FHS**	**CIQ NRS**	**GPE**	**AUC**
FHS	27.1 (20.8)	-0.67	-	-0.79	0.72	-0.73	-0.52	0.80
NRS-child	-31.1 (25.7)	0.66	-0.79	-	-0.73	0.82	0.46	0.77

^a^spearman’s rho.

^b^correlation to mean of scores of included subscales of CHQ-PF50.

^c^scales of OM-6.

### Minimal important change (MIC)

**Figure 1:**
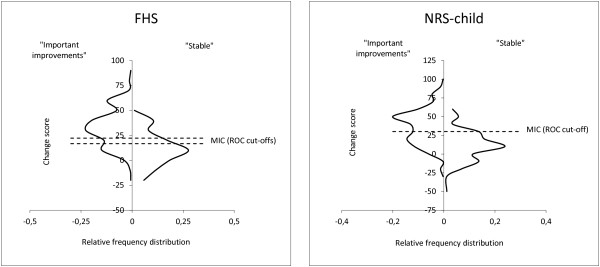
**Responsiveness and minimal important change - distribution of change scores after dichotomizing children in groups of “stable” versus “importantly improved”.** Change scores were rounded to the nearest 10 before plotting for visual enhancement.

Table 8 presents results on ROC cut-off points with specificity and sensitivity. MIC was smaller than SDC at the individual level for the FHS scale when choosing a cut-off of 16.7 as a benchmark for the MIC. However, MIC will be beyond measurement error in groups of two or more ((19.8/16.7)^2^). Figure 1 presents the distribution of change scores related to the anchor.

**Table 8:** Smallest detectable change and minimal important change

	**Range**	**SDC**	**MIC**	**Sensitivity**	**Specificity**
FHS	0-100	19.8	16.7	0.8	0.7
			22.2	0.7	0.8
NRS-child	0-100	25.9	30.0	0.7	0.7

## Discussion

OM-6 is now available in a reliable, valid, responsive and interpretable Danish version. Analysis of especially structural validity revealed issues that must be considered in the application of the instrument. Confirmatory factor analysis did not confirm the initial hypothesized one-factor model of the FHS scale. A superior fit was achieved after allowing for covariance between item 2 (hearing loss) and item 3 (speech impairment). It has good test-retest reliability and construct validity, is able to discriminate between relevant subgroups and is responsive to change. Furthermore, this study is the first to present results on the SDC and patient perceived MIC.

### Translation and cross-cultural adaptation of OM-6

To enhance cross-cultural equivalence a thorough translation and cultural adaption procedure was performed [14]. The process revealed several issues important to the Danish language and culture and we recommend these issues to be considered in cultures similar to the Danish. OM-6 has previously been translated into Dutch. However, none of these studies elaborate further on possible issues in the translational process [9,11]. Future studies should include this important aspect of the cross-cultural adaption to ensure optimal content validity.

### Validity

We performed confirmatory factor analysis to test if the FHS scale satisfied a one-factor structure measuring functional health in children with otitis media. The initial analysis did not produce an acceptable fit, however, by allowing covariance between item 2 (hearing loss) and item 3 (speech impairment) we obtained a superior fit. This makes sense conceptually as hearing and speech development are closely connected especially in this age group. Internal consistency was acceptable. However, analysis revealed items 2 and 3 to have considerably lower item-total correlations than the remaining items. Some issues need further discussion to explain these findings. First, the age of the patient population may be an issue. Feedback from respondents indicated that it was difficult for caregivers to evaluate speech impairment (item 3) as most of the children in this study sample are very young and still only in the early stages of language development. Furthermore, the accuracy of caregiver’s perceptions of the child’s hearing (item 2) is questionable. The developers of OM-6 addressed this issue by correlating scores of item 2 (hearing loss) with audiometric findings. They concluded that the caregiver’s perceptions of hearing for children were less than accurate [36]. Despite the possible deficiencies of items 2 and 3 we still believe that they have good face validity as both cover fundamental concerns in otitis media. Second, one may question if OM-6 consists of more than one factor. It is possible that items 2 and 3 comprise an individual factor which may, in part, explain the low item-total correlations of these items. We did not explore this option further because of the brevity of the instrument and the acceptable CFA fit in the one-factor model including a single covariance. Based on the findings of this study we recommend that the original structure of the OM-6 is maintained but encourage future studies to continue exploring the factorial structure of the OM-6 in different study populations diagnosed with OM.

In a subgroup analysis we found that the final model fitted well on data from the only-OME children but the fit was not optimal for data from children diagnosed with rAOM (with or without OME). We had to allow for an additional covariance between item 4 (emotional distress) and item 6 (caregiver concerns) in order to obtain an acceptable fit. The finding of large correlation between error terms of these items also makes conceptual sense as children experiencing rAOM are likely to present a more severe clinical picture with recurrent fever and pain. This often leads to considerable emotional distress in the child which will add to caregiver concerns. The need for an additional modification to the model suggests that OM-6 may be more suited for children with OME rather than children who experience rAOM alone or in connection with OME. However, children referred for tympanostomy tube insertion due to severe rAOM (with or without OME) tend to be younger than children referred because of only OME. Hence, it becomes difficult to distinguish the impact of diagnosis from the impact of age when explaining this finding. That said, it is important to acknowledge the possible weaknesses pointed out in this study when utilizing this instrument.

Strong construct validity was found by testing hypothesized correlations (87.5% were correctly predicted). Furthermore, both scales were able to discriminate between subgroups when dichotomizing in children suffering from rAOM and no rAOM. In general, our results on construct validity are consistent with those found in the literature [5,9-11]. However, direct comparisons between results are difficult due to the heterogeneity of methods used for assessing construct validity.

### Reproducibility

Test-retest reliability (ICCs) was acceptable and similar to previously published results [11]. Agreement between test-retest scores was investigated by assessment of the systematic and measurement error (Table 6). The measurement error (SDC) of the FHS scale was 19.8 and 25.9 for the NRS scale. This indicates that a change of less than one fifth (FHS) and one fourth (NRS) is within normal scale variability. Measurement error and test retest reliability is highly dependent on the stability of the study sample from which the data is obtained and one may question if the measurement error is due to “instability” of the study sample rather than scale variability. We do not believe this to be the case as ICCs were acceptable and systematic error is negligible. Furthermore, our findings on measurement error correspond to findings published by Brouwer *et al.*[11]. However, the magnitude of the measurement error indicates that using the OM-6 on individual patients may be problematic as change scores have to be large before they can be relied upon. We recommend using the OM-6 at group level as measurement error decreases with the square root of N [26].

### Responsiveness

No floor/ceiling effects were found enabling bidirectional change scores in longitudinal studies. Construct responsiveness revealed strong correlations between change scores of the different instruments (Table 7). Similarly, criterion responsiveness showed acceptable results although correlations between the GPE scale and the NRS scale were less strong. This is in concordance with other studies reporting on the responsiveness of the OM-6 [5,9-11]. Three studies [5,9,10] present standardized response means (SRM) above 1.0 and conclude that OM-6 is responsive to change [27,37]. The last study [11] concluded that OM-6 was responsive to change using Guyatt’s Responsiveness statistic (GRS) and statistical significance of differences between scores at the different time points. We did not apply any of these methods as neither SRM, GRS or statistical significance between scores provide information regarding the validity of change scores [23].

### Minimal important change (interpretability)

When interpreting OM-6 change scores, it is not only important to know whether results are statistically significant, but also whether they are relevant for children/caregivers or clinicians. By including the GPE-scale we aimed to assess the MIC as perceived by the patients. However choosing a ROC cut-off point as a parameter of MIC is highly dependent on the aim of the investigation. Often the aim will be to assess change after intervention. In this situation we recommend a cut-off value of 16.7 on the FHS as true-positives may be more important than true-negatives. This cut-off is within measurement error (SDC) and sample size should be adjusted accordingly in order to minimize measurement error. However, results of this study show that MIC will be beyond SDC even in very small groups. On the other hand, if the primary objective is to distinguish between “importantly improved” and “stable” children a cut-off value of 22.2 will be the most appropriate as this represents the optimum cut-off when weighting sensitivity and specificity equally. Two other studies have commented on the interpretability of change scores on the OM-6. In the original article on OM-6, Rosenfeld *et al.* proposed guidelines adopted from a study on asthma patients [5,38]. Brouwer *et al.* presented results on “minimally clinical important difference” (MCID) by applying distribution and anchor-based methods [11]. For the distribution based methods, MCID was calculated using effect sizes (ES) and standard error of measurement (SEM) as benchmarks. ES and SEM are both statistical parameters linked to the measurement variance (error) and therefore refer more to the SDC than the MIC [23]. This is also supported by the fact that both estimates correspond well with the SEM of our study. For the anchor based methods calculations of MCID was based on anchors reflecting important differences perceived by clinicians rather than patients/respondents. Hence, results of this study are not directly comparable with those presented by Brouwer *et al.* as we wanted to investigate important change as perceived by the patients. The clinical picture of rAOM and OME may be very different from patient to patient and severity of symptoms may also vary greatly between episodes. Thus, there may be discrepancy between what clinicians and respondents perceive as important change. Therefore, we believe that in order to specifically investigate the impact of rAOM and OME on these children and their families, it is important to include anchor-questions aimed directly at this issue e.g. by applying a GPE-scale.

### Limitations of this study

This study was conducted on a subgroup of children suffering from OM, as indication for ventilating tube treatment had to be present. Therefore, if these translations are to be applied in more heterogeneous populations basic investigations regarding reliability and validity should be conducted as for example floor/ceiling effects may be present in populations with less severe OM.

Questionnaires were administered differently for the assessment of test-retest reliability. Pre-baseline questionnaires were completed on paper in the ENT clinic and baseline questionnaires were completed at home. Differences between the test-retest scores may, in part, be explained by an intention to give socially desirable answers at the clinic or being more distracted by external factors e.g. by a crying or impatient child. Furthermore, 82% of caregivers chose to complete baseline questionnaires electronically. Although the wording was the same, layout differences were inevitable which may also have contributed to differences between pre-baseline and baseline scores. As our results on reliability and agreement were in accordance with similar studies we believe this factor to be negligible.

A GPE-scale was applied for the analysis of criterion responsiveness and the assessment of MIC. However, Although GPE scales have been found to be reliable and valid measures of health transition [39], this study is weakened by the fact that we did not assess the psychometric properties of the scale beyond correlations to baseline, follow up and change scores. Furthermore, these analyses revealed issues that need mentioning. Firstly, correlation to change scores was only moderate for the NRS scale. Secondly, correlations with baseline and follow up scores revealed that scores on the GPE scale were strongly influenced by the current status (results not presented). This is in concordance with findings in other studies [39,40]. Therefore, the MICs presented in this study should only be regarded as an indication to what the caregivers perceive as important change. Further studies on the MIC for the OM-6 are warranted.

## Conclusion

The Danish version of the OM-6 is a reliable, valid, responsive and interpretable questionnaire to measure quality of life in young children with otitis media. Our results highlight possible weaknesses of the instrument that needs to be acknowledged when utilizing OM-6. Despite these issues our analysis supports the continuing use of OM-6 in studies on populations with otitis media.

## Consent

By completing the questionnaires consent from the patient’s guardian/parent/next of kin was implied for the publication of this report and accompanying images. All participants received thorough study information by letter and telephone at inclusion.

## Abbreviations

AOM: Acute otitis media; AUC: Area under curve; CHQ-PF50: Child Health Questionnaire 50 item version; CFA: Confirmatory factor analysis; CFI: Comparative fit index; CIQ: Caregiver impact questionnaire; d.f.: Degrees of freedom; EPC: Expected parameter changes; ES: Effect size; FHS: Functional health status; GPE: Global perceived effect; GRS: Guyatt’s responsiveness statistic; ICC: Intraclass correlation coefficient; Iqr: Interquartile range; MCID: Minimally clinical important difference; MI: Modification index; MIC: Minimal important change; NRS: Numerical rating scale; OM: Otitis media; OME: Otitis media with effusion; OM-6: Otitis Media 6 questionnaire; QoL: Quality of life; rAOM: Recurrent acute otitis media; ROC: Receiver operating characteristics; RMSEA: Root mean square error of approximation; SD: Standard deviation; SDC: Smallest detectable change; SEM: Standard error of measurement; SRM: Standardized response mean; SRMR: Standardized root mean square residual.

## Competing interests

The authors declare that they have no competing interests.

## Authors’ contributions

CH, HL and CG wrote the study protocol. CH administered the questionnaires, collected and analysed the data and wrote the manuscript. HL participated in analysis and interpretation of the data. EJ helped with inclusion of patients. AK and CF participated in the design of the study. All Authors critically revised the manuscript and gave final approval of the manuscript.

## Authors’ information

Christian Godballe, Anette Drøhse Kjeldsen, Eva Charlotte Jung Johansen, Christian Emil Faber and Henrik Hein Lauridsen are co-authors.

## Supplementary Material

Additional file 1 Measurement instruments, construct validity hypotheses and test-retest group analysis. Click here for file
