# Supplementary material for: The Otitis Media-6 questionnaire: psychometric properties with emphasis on factor structure and interpretability
Source: Health Qual Life Outcomes. 2013 Nov 20;11:201. doi: 10.1186/1477-7525-11-201 (PMC4222717; doi:10.1186/1477-7525-11-201)
Supplement: Additional file 1 — Measurement instruments, construct validity hypotheses and test-retest group analysis. [file 1477-7525-11-201-S1.docx]

Additional file 1

Measurement instruments

Questionnaires/subscales and single questions included in the analyses:

**OM-6**

FHS scale

**Physical suffering:** Ear pain, ear discomfort, ruptured ear drum, high fever, or poor balance. How much of a problem for your child during the past 4 weeks?

Response options: 1) not present/no problem, 2) Hardly a problem at all, 3) somewhat of a problem, 4) moderate problem, 5) quite a bit of a problem, 6) very much a problem, 7) extreme problem

**Hearing loss:** Difficulty hearing, questions must be repeated, frequently says "What?", or television is excessively loud. How much of a problem for your child during the past 4 weeks?

Response options: 1) not present/no problem, 2) Hardly a problem at all, 3) somewhat of a problem, 4) moderate problem, 5) quite a bit of a problem, 6) very much a problem, 7) extreme problem

**Speech impairment:** Delayed speech, poor pronunciation, difficult to understand, or unable to repeat words clearly. How much of a problem for your child during the past 4 weeks?

Response options: 1) not present/no problem, 2) Hardly a problem at all, 3) somewhat of a problem, 4) moderate problem, 5) quite a bit of a problem, 6) very much a problem, 7) extreme problem

**Emotional distress:** Irritable, frustrated, sad, restless, or poor appetite. How much of a problem for your child during the past 4 weeks because of ear infections or fluid?

Response options: 1) not present/no problem, 2) Hardly a problem at all, 3) somewhat of a problem, 4) moderate problem, 5) quite a bit of a problem, 6) very much a problem, 7) extreme problem

**Activity limitations:** Playing, sleeping, doing things with friends/family, attending school or day care. How limited have your child's activities been during the past 4 weeks because of ear infections or fluid?

Response options: 1) not limited at all, 2) Hardly limited at all, 3) very slightly limited, 4) slightly limited, 5) moderately limited, 6) very limited, 7) severely limited

**Caregiver concerns:** How often have you, as a caregiver, been worried, concerned, or inconvenienced because of your child's ear infections or fluid over the past 4 weeks?

Response options: 1) none of the time, 2) Hardly any time at all, 3) a small part of the time, 4) some of the time, 5) a good part of the time, 6) most of the time, 7) all of the time

NRS-child scale

0verall, how would you rate your childs quality of life as a result of ear infection or fluid?

Response options: Scale range 0-10, 0) worse possible quality of life, 5) Half-way between worst and best, 10) best possible quality of life

**CIQ**

FHS scale

How often did you or your partner experience the following problems during the past 4 weeks as a consequence of ear infections or ear fluid in your child? Please circle one number for each question.

1. Lack of sleep

2. Absence from work or education

3. Cancelling of family activities, such as trips, play dates, vacations

4. Changing daily activities, such as housework, shopping, or time with other siblings

5. Feeling nervous, agitated, or irritable

6. Feeling helpless or frustrated

Response options for all of the above: 1) none of the time, 2) hardly any of the time, 3) a little of the time, 4) some of the time, 5) a good bit of the time, 6) most of the time, 7) all of the time.

NRS-caregiver scale

Overall, how would you rate your quality of life during the past 4 weeks as a result of your child's ear infections or fluid?

Response options: scale range 0-10, 0) worse possible quality of life, 5) Half-way between worst and best, 10) best possible quality of life

**CHQ-PF50 (**List of subscales included in the analyses)

Global Health (GGH)

In general, would you say your child’s health is?

Role/Social limitations – Physical (RP)

During the past four weeks, has your child’s school work or activities with friends been limited in any of the following ways due to problems with his/her PHYSICAL HEALTH?

Bodily Pain/Discomfort (BP)

During the past four weeks, how much bodily pain or discomfort has your child had?

During the past four weeks, how often has your child had bodily pain or discomfort?

Mental Health (MH)

During the past 4 weeks, how much of the time do you think your child: **a.** felt like crying? **b.** felt lonely? **c.** acted nervous? **d.** acted bothered or upset? **e.** acted cheerful?

General Health Perceptions (GH)

How true or false is the statement for your child? **a.** My child seems to be less healthy than other children I know. **b.** My child has never been seriously ill. **c.** When there is something going around my child usually catches it. **d.** I expect my child will have a very healthy life. **e.** I worry more about my child’s health than other people worry about their children’s health.

Parental Impact – Emotional (PE)

During the past 4 weeks, how MUCH emotional worry or concern did each of the following cause YOU : **a.** Your child’s physical health **b.** Your child’s emotional well-being or behavior **c.** Your child’s attention or learning abilities

Parental Impact – Time (PT)

During the past 4 weeks, were you limited in the the amount of time YOU had for your own personal needs because of: **a.** Your child’s physical health **b.** Your child’s emotional well-being or behavior **c.** Your child’s attention or learning abilities

4-week history questions:

Number of days of antibiotic use?

Number of visits to the doctor?

Number of interrupted nights?

Number of days observed lower activity level in the child?

**GPE scale**

Overall, how would you rate your child’s quality of life as a result of middle ear infection or fluid in the middle ear now compared to how it was before tube treatment?

Response options: 1) very much improved, 2) much improved, 3) a little improved, 4) no change, 5) a little deterioration, 6) much deterioration, 7) very much deterioration.

Construct validity

Tables contain construct validity hypotheses. A correlation of <0.3 was defined as weak, 0.3-0.5 as moderate and >0.5 as strong.

| **OM-6** | **CHQ-PF50** | | | |
| --- | --- | --- | --- | --- |
| Question | Subscale | predicted corr. | Comment | obtained corr. |
| Physical suffering (FHS) | BP | Strong neg. | If pain is present in OM, it will also become apparent in more generalized questions regarding bodily pain. | **-0.82** |
|  |  |  |  |  |
| Emotional distress (FHS) | MH | Strong neg. | If OM has emotional impact on the child this will become apparent in general questions regarding emotional well-being as well. | **-0.66** |
| Activity limitations (FHS) | RP | Moderate neg. | RP subscale and item 5 of the OM-6 covers the same construct. However, item 5 is more extensive. | **-0.56** |
|  |  |  |  |  |
| Caregiver concern (FHS) | PE | Moderate neg. | It may be hard to determine attention or learning abilities in children below 2-3 years of age. | **-0.49** |
|  | PT | Moderate neg. | It may be hard to determine attention or learning abilities in children below 2-3 years of age | **-0.57** |
| NRS-child | GGH | Moderate neg. | Although not measuring the same, the constructs “global health” is likely to be affected by “disease specific QoL” | **-0.33** |
|  | GH | Moderate neg. | Although not measuring the same, the constructs “general health” is likely to be affected by “disease specific QoL” | **-0.40** |
|  | Summary score of CHQ-PF50 subscales | Strong pos | The summary score should correlate strongly to the overall disease specific HRQoL of the child because OM is thought to have significant impact on the general well-being of the child | **0.73** |
| OM-6 FHS summary score | Summary score of CHQ-PF50 subscales | Strong neg | The summary score should correlate strongly to the summary score of the OM-6 FHS subscale because OM is thought to have significant impact on the general well-being of the child | **-0.74** |

| **OM-6** | **4 week history questions** | | | |
| --- | --- | --- | --- | --- |
| Question | Question | predicted corr. | comment | obtained corr. |
| Physical suffering (FHS) | No. of days of antibiotic use | Moderate  Pos. | Will to some extend be correlated to pain, although pain may be present without indication for ant. Treatment. | **0.44** |
|  | No. of visits to the doctor | Moderate pos. | Same as above. | **0.55** |
| Activity limitations (FHS) | No. of interrupted nights | Strong pos. | Only partially covers item 5, but parents are thought to focus on sleep | **0.58** |
|  | No. of days observed lower activity level in the child | Strong pos. | Both items regard the child´s activity level, although OM6 item is more extensive. | **0.65** |

| **OM-6** | **OM-6 FHS item/summary score or NRS-child** | | | |
| --- | --- | --- | --- | --- |
| Question | Question | predicted corr. | comment | obtained corr. |
| Hearing loss | Om6 FHS item 3 (speech impairment) | Strong pos. | Hearing and language should be strongly correlated | **0.59** |
| NRS-child | OM6 FHS summary score | Strong neg. | FHS should correlate strongly to QoL | **-0.77** |

| **OM-6** | **CIQ FHS items/summary score or NRS-caregiver** | | | |
| --- | --- | --- | --- | --- |
| Question | Question | predicted corr. | comment | obtained corr. |
| Activity limitations | CIQ FHS (Lack of sleep) | Strong pos. | Parents are thought to focus on sleeping. | **0.72** |
| Caregiver concern | CIQ FHS (Cancelling of family activities, such as trips, play dates, vacations) | Strong pos. | Cancelling of family activities should be related strongly to inconvenience of the parent | **0.55** |
|  | CIQ FHS (Changing daily activities, such as housework, shopping, or time with other siblings) | Strong pos. | Changing daily activities should be related strongly to inconvenience of the parent | **0.63** |
|  | CIQ FHS Summary score | Strong pos. | Summary score of impact on caregiver FHS (CIQ FHS) is strongly related to caregiver concern (same construct). | **0.79** |
|  | NRS-caregiver | Strong neg. | Overall disease specific caregiver HRQoL is strongly related to caregiver concern. | **-0.75** |
| NRS-child | NRS-caregiver | Strong pos. | Strong correlation between child and caregiver HRQoL as OM in the child is thought to have impact on the whole family. | **0.83** |
|  | CIQ FHS summary score | Strong neg. | Strong correlation between child HRQoL and caregiver FHS as OM in the child is thought to have impact on the whole family. | **-0.75** |
| OM-6 FHS summary score | CIQ FHS summary score | Strong pos. | Strong correlation between child and caregiver FHS as OM in the child is thought to have impact on the whole family. | **0.72** |
|  | NRS-caregiver | Strong neg. | Strong correlation between child FHS and caregiver HRQoL as OM in the child is thought to have impact on the whole family. | **-0.71** |

Test-retest group

Table presents comparison analysis between the test-retest group and the remaining study population – no significant differences on variable age, gender, diagnostic subgroup or baseline scores.

|  | Test-retest group (N=135) | Remaining study population (N=300) | p-value |
| --- | --- | --- | --- |
| Age, median (Iqr) | 1.42 (1.02) | 1.46 (1.28) | 0.523 ^a^ |
| Gender male, N (%) | 80 (54.7) | 164 (59.3) | 0.372 ^a^ |
| Diagnostic subgroup, N (%) | OME: 67 (49.6)  rAOM: 22 (16.3)  rAOM/OME: 45 (33.3) | OME: 139 (46.3)  rAOM: 41 (13.7)  rAOM/OME: 118 (39.3) | 0.242 ^b^ |
| Baseline FHS score, mean (SD) | 42.9 (18.7) | 45.2 (19.0) | 0.252 ^c^ |
| Baseline NRS score, mean (SD) | 50.8 (23.2) | 49.0 (23.2) | 0.518 ^a^ |

^a^ Wilcoxon rank-sum (Mann-Whitney), ^b^ one-way analysis of variance (ANOVA), ^c^ students t-test
